# Which Antibody Functions are Important for an HIV Vaccine?

**DOI:** 10.3389/fimmu.2014.00289

**Published:** 2014-06-18

**Authors:** Bin Su, Christiane Moog

**Affiliations:** ^1^INSERM U1109, Fédération de Médecine Translationnelle de Strasbourg, Université de Strasbourg, Strasbourg, France

**Keywords:** HIV, mucosal HIV vaccine, cell-to-cell transfer, neutralizing antibodies, non-neutralizing inhibitory antibodies, FcγR, antigen-presenting cells, ADCC

## Abstract

HIV antibody (Ab) functions capable of preventing mucosal cell-free or cell-to-cell HIV transmission are critical for the development of effective prophylactic and therapeutic vaccines. In addition to CD4^+^ T cells, other potential HIV-target cell types including antigen-presenting cells (APCs) (dendritic cells, macrophages) residing at mucosal sites are infected. Moreover, the interactions between APCs and HIV lead to HIV cell-to-cell transmission. Recently discovered broadly neutralizing antibodies (NAbs) are able to neutralize a broad spectrum of HIV strains, inhibit cell-to-cell transfer, and efficiently protect from infection in the experimentally challenged macaque model. However, the 31% protection observed in the RV144 vaccine trial in the absence of detectable NAbs in blood samples pointed to the possible role of additional Ab inhibitory functions. Increasing evidence suggests that IgG Fcγ receptor (FcγR)-mediated inhibition of Abs present at the mucosal site may play a role in protection against HIV mucosal transmission. Moreover, mucosal IgA Abs may be determinant in protection against HIV sexual transmission. Therefore, defining Ab inhibitory functions that could lead to protection is critical for further HIV vaccine design. Here, we review different inhibitory properties of HIV-specific Abs and discuss their potential role in protection against HIV sexual transmission.

## Introduction

Sexual transmission is currently the major route of HIV infection worldwide. In more than 80% of newly diagnosed cases of HIV-1 infection, the patients become infected during sexual intercourse (1). This route of infection can be prevented by IgG neutralizing antibodies (NAbs) and secretory IgA (2, 3). Recently discovered potent and broadly NAbs (bNAbs) are able to neutralize a broad spectrum of cell-free and cell-associated HIV strains (4–13). These antibodies (Abs) have also been shown to efficiently protect non-human primates (NHP) and humanized mice from experimental challenge (14–20). However, bNAbs display very specific characteristics and are extremely difficult to induce since only 10–30% of patients develop such Abs (21–25) and attempts to induce them by vaccination have failed. bNAbs are characterized by uncommonly long complementarity-determining loops and extensive somatic hypermutation, suggesting the need for a long maturation process, which makes their induction by vaccination extremely difficult.

Interestingly, the limited 31% protection observed in the RV144 vaccine trial in the absence of detectable NAbs in plasma/serum specimens pointed to a possible role of additional Ab inhibitory functions in this protection (26, 27) and defining these additional functions is therefore critical. Increasing evidence suggests that IgG Fcγ receptor (FcγR)-mediated inhibition of Abs, leading to phagocytosis or antibody-dependent cellular cytotoxicity (ADCC), plays a role in protection. These FcγRs are expressed on various antigen-presenting cells (APCs) and natural killer (NK) cells present at the mucosal site, suggesting that Fc-mediated inhibitory functions may contribute to the blockage of mucosal transmission. These cells may play a decisive role during sexual transmission since they have been proposed to be the first HIV targets at the mucosal site (28–30). Evidence from *in vivo* studies showed that HIV-specific Abs displaying Fc-mediated inhibition in the absence of neutralizing activity is able to decrease the viral load after experimental vaginal challenge in the macaque model (31, 32). Besides, various Ab inhibitory functions at the mucosal site such as aggregation, complement inhibition, inhibition of HIV transfer, and inhibition by induction of antiviral cytokines and chemokines may also contribute to HIV protection. In addition to the induction of NAbs, new vaccination strategies based on such Ab activities, should be considered. In the present review, HIV inhibition by Abs based on these various potential inhibitory functions will be discussed, as well as its possible contribution to the development of new vaccination strategies.

## HIV-1 Transmission through Mucosal Tissues

**Figure 1:**
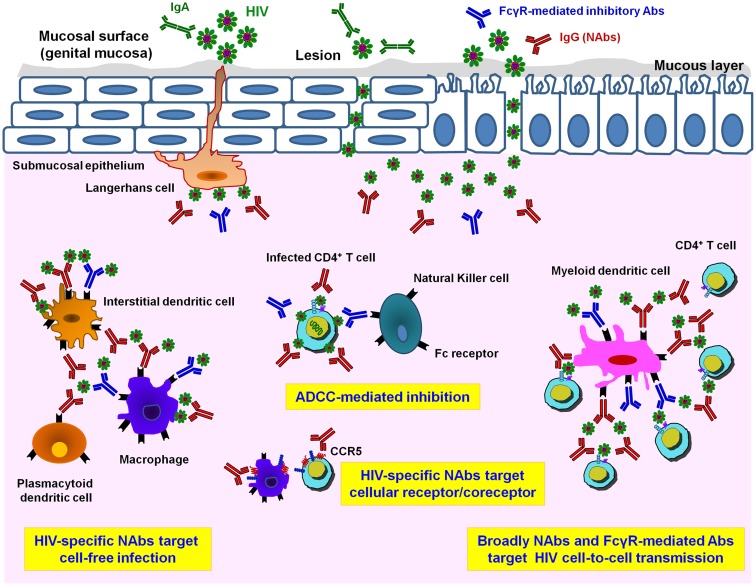
**Different HIV-1 antibody activities in mucosal tissues**. Infectious HIV-1 particles can cross a multi-cellular layer of stratified squamous epithelial cells in genital mucosal tissues. Both cell-free and cell-associated HIV-1 virions infect host cells. Langerhans cells transport the virus into the sub-epithelium and mucosal lesions may provide an accessible pathway for HIV-1. In the sub-epithelium, in addition to target CD4^+^ T cells, other potential HIV-target cell types including myeloid dendritic cells (DCs) and macrophages are infected either by cell-free virions or by cell-associated virions. Mucosal HIV-specific IgA (IgA, in green) can bind and neutralize cell-free virus at mucosal surfaces. Adaptive immune responses such as HIV-1-specific IgG neutralizing antibodies (NAbs, in red) are important for preventing HIV-1 cell-free infection. Only NAbs are able to inhibit HIV infection of CD4 T lymphocytes while both NAbs and FcγR-mediated inhibitory Abs (in blue) help to inhibit the spread of infection *via* cell-to-cell transmission route. Prevention of HIV-1 infection and killing of virus-producing cells by Ab-dependent mechanisms, especially antibody-dependent cellular cytotoxicity (ADCC) *via* binding of Fc receptors presented on the surface of innate immune cells such as natural killer (NK) cells, monocytes, DCs, or macrophages, takes place by inhibiting viral replication and diminishing viral reservoirs *in vivo*. Moreover, inhibitory NAbs directed to cellular target epitopes, such as CCR5 or other HIV-receptor/co-receptor structures, could provide additional targets for the rational design of novel vaccine candidates.

Very little is known about how HIV infects and disseminates through mucosal tissues. The selection of transmitted/founder (T/F) virus occurs at the mucosal portal of HIV entry (33–38). Mucosal sites contain a variety of immune cells targeted by HIV, i.e., APCs comprising various types of dendritic cells (DCs), macrophages, NK cells, and CD4 T lymphocytes (28–30, 39–43) (Figure 1). However, the exact mechanism by which viral particles migrate through the epithelial barrier remains unclear. Various modes of infection have been proposed, which include transfer through epithelial cells and intestinal epithelium, transport of HIV *via* DCs present at mucosal surfaces, and direct infection of resident CD4 T cells (41, 44–48) (Figure 1). Apart from direct infection of immune cells by cell-free virus, cell-to-cell transmission has been suggested to play a major role in HIV propagation and dissemination *in vivo*. Spread of HIV infection by cell-to-cell transmission has been found to be 100- to 1000-fold more efficient than infection by cell-free virions (49–54). At the mucosal level, in addition to CD4 T cells many cells are targeted by direct cell-free or cell-associated HIV-1 and the inhibition of these multiple routes of infection involve numerous immunological defenses (55), such as secretory IgA aggregation, Fc-mediated inhibition, neutralization of CD4 T cell infection, lysis of infected cells by NK cells, phagocytosis after antigen presentation, and inhibition following cytokine and chemokine production (Figure 1). For example, HIV-1 trapped by DCs can be inhibited by Fc-mediated inhibitory Abs, whereas inhibition of HIV-1 transfer from DCs to T cells will involve potent HIV NAbs (56). Therefore, in addition to neutralization of HIV-1-infected CD4 T cells by specific bNAbs, numerous additional inhibitory pathways, depending on the amount and type of HIV-1 and on the type of cells in the mucosa, may participate in HIV-1 inhibition and could decrease the concentration of NAbs necessary for protection.

## Mucosal B-Cell Responses

A major defensive mechanism from the mucosal immune system involves local production and secretion of IgG and dimeric or multimeric IgA from B-cells (57–60). The initial immune stimulation occurs mainly in mucosa-associated lymphoid tissues, particularly Peyer’s patches of the distal ileum and other parts of gut-associated lymphoid tissues (61, 62). From these inductive sites the activated B-cells reach peripheral blood by migrating through lymph and draining lymph nodes and subsequently extravagate at secretory effector sites on a competitive basis depending on complementary adhesion molecules and chemokine–receptor pairs (61, 63). In addition, B-cells with “innate-like” functions including B-1 cells are enriched in mucosal tissues and marginal zone B-cells (64). These B-cells produce natural Abs that recognize conserved features of bacterial carbohydrates and phospholipids, that generate a first line of protection through the early production of low-affinity IgM in response to bacteria (62, 64–66). Mucosal DCs support B-cell activation and several factors in mucosal tissues, including both T cell-dependent and T cell-independent factors have been show to favor B-cell immunoglobulin class-switching to IgA-secreting plasma cells (59, 67). However, the exact local production sites and local redistribution at the mucosal site have not been well documented. During acute HIV infection phase, naïve B-cells are immediately decreased and reciprocal memory B-cell increased at mucosal sites and blood although little is known on the phenotypic features and functions of B-cell populations and early B-cell subversions occurring at mucosal sites (68). As most HIV-1 transmission occurs *via* mucosal sites, eliciting effective mucosal B-cell responses with long-lasting protective NAbs at mucosal sites is therefore critical to provide the first line of protection at mucosal surfaces for preventing early HIV-1 invasion by HIV-1 vaccine (69–71).

## Mechanisms of Inhibitory Activity of Neutralizing Antibodies

**Figure 2:**
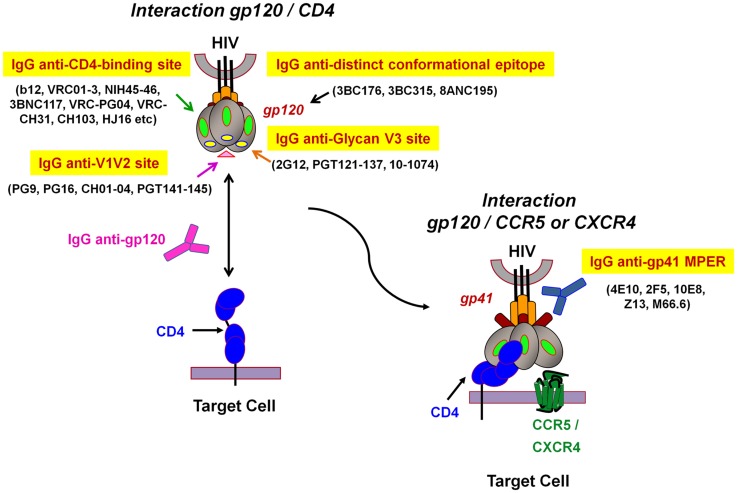
**Model representation of HIV-1 envelope glycoprotein structure and epitopes of broadly neutralizing antibodies**. The surface receptor binding subunit gp120 and the fusion-mediating transmembrane subunit gp41 make up the functional HIV-1 envelope glycoproteins. The targets of broadly neutralizing antibodies (bNAbs) can be divided into several groups: (1) IgG anti-CD4-binding site, (2) IgG anti-V1V2 site, (3) IgG anti-*N*-linked glycan V3 site, and (4) IgG anti-gp41 membrane proximal external region (MPER). The IgG anti-distinct conformational epitope present on the envelope trimer, remains to be determined (adapted from Dr. Béatrice Labrosse).

Most HIV-1 vaccination strategies aim to induce human HIV-specific Abs able to inhibit the infection of target cells at the onset of viral transmission (2, 11, 72). Humoral responses against HIV have been extensively studied and NAbs able to efficiently neutralize *in vitro* a broad range of circulating HIV-1 strains have been described (10, 12, 20). These include the well-characterized NAb b12, 2G12, 447-52D, 2F5, 4E10, as well as novel bNAbs such as VRC01 and 10–1074 or belonging to PGT family that neutralize a large spectrum of HIV-1 isolates of various clades (4–7, 12, 73–75) (Figure 2). These Abs efficiently inhibit cell-free HIV primary isolates or pseudoviruses *in vitro* in conventional neutralization assays with peripheral blood mononuclear cells (PBMCs) or HIV-permissive cell lines (TZM-bl). Both assays assess the capacity of Abs to inhibit HIV-1 infection of either CD4^+^ primary cells or TZM-bl cell lines that express the CD4 receptor and co-receptor CCR5. Abs possessing a neutralizing activity will recognize functionally important structures and conserved epitopes of the HIV viral envelope gp120 and gp41, and will impede virus attachment as well as fusion and entry processes that lead to a decrease in HIV replication (9–12) (Figure 2). The neutralization process is due to the capacity of Abs to directly inhibit HIV-1 replication in the absence of additional factors, such as Fc receptors (FcRs) or complement. Yet, due to the complex glycosylation profile of HIV and conformational changes of the viral envelope during fusion (Figure 2), most NAbs require long HCDR3s to allow the recognition of poorly accessible conserved Env epitopes (76). Moreover, NAbs isolated from infected patients result from a long maturation and somatic hypermutation processes (9–12). These unusual Ab characteristics will unfortunately be extremely difficult to generate by vaccination. Several of these HIV-1 bNAbs have been reverted experimentally to their unmutated ancestral state, and were found to bind weakly or undetectably to native HIV-1 Env (77, 78), which means that Ab responses induced by vaccination will have to occur following intricate pathways of B-cell maturation.

Recent studies showed that high levels of IgG Abs specific for the first and second variable regions (V1V2) of gp120 were inversely associated with a reduced risk of HIV-1 infection in the RV144 clinical vaccine trial (27, 79–81). Moreover, Yates et al. recently found that vaccine-induced HIV-1-specific IgG3 responses correlated with decreased risk of infection in RV144 clinical trial compared to the VAX003 vaccine regimen (82). Since partial protection observed in the RV144 phase III Thailand trial was mediated by the induction of non-neutralizing antibodies (NNAbs) and a moderate T cell response (27, 83), it seems that other immune mechanisms in addition to classical NAbs responses are required to achieve protection against HIV infection.

## Mechanisms of Fc Receptor-Mediated Protection

The FcR-dependent mechanism of inhibition has been observed in various HIV-target cells that express these receptors, for instance DCs, Langerhans cells, and macrophages (56, 84–90). HIV inhibition involving interactions with FcR receptors was confirmed with the cell line TZM-bl that expresses various FcγRs (91, 92) mainly FcγRI and FcγRII (84, 85, 91). Fc-mediated inhibition increased by 10- to 1000-fold the inhibitory activity of NAbs in FcR-bearing macrophages (84), and neutralization titers of NAbs 4E10 and 2F5 were increased as much as 5000-fold in the case of TZM-bl cells expressing FcγRI (91, 92). Some HIV-1-specific Abs lacking neutralizing activities have also been shown to display Fc-mediated inhibitory activities (93). Such Abs, which inhibit HIV-1 replication only *via* FcγR receptors are referred to as non-neutralizing inhibitory Abs (NNIAbs) (85). In the case of APCs bearing FcγR, the formation of immune complexes between Abs and HIV leads to phagocytosis of the virus and its degradation by specific lysosomes (88, 94–96). Moreover, the fixation of Abs on the FcR of effector cells can also induce antiviral cytokines and chemokines, further impeding viral replication (97, 98). The mechanism of inhibition of NNIAbs implies that, contrary to NAbs, they do not need to recognize functional Env spikes. NNIAbs capture the virus *via* the Fab domains and bind to FcR-bearing cells *via* their Fc domains, increasing therefore the number of potential epitopes susceptible to be targeted by immunogens.

Recently, it has been shown that V1V2-specific IgG3 subclass Abs are associated with broad antiviral responses and were correlated with a decreased risk of infection in the RV144 vaccine trial (82). Chung et al. also found that in this trial, NNAbs were induced that presented highly coordinated Fc-mediated effector responses by the selective induction of highly functional IgG3 (99). These studies indicate that functional activity and Ab subclass may contribute to the potential antiviral activity of Abs that extends beyond virus neutralization and illustrate the potential role of FcγR-mediated innate and adaptive immune functions in additional HIV-1 protective mechanisms.

## Antibody-Dependent Cellular Cytotoxicity

Antibody-dependent cellular cytotoxicity was reported in HIV-infected patients in 1987, when it was shown that HIV envelope gp120 bound to CD4 T cells was sensitive to lysis by PBMCs from HIV-infected patients (100). This ADCC mechanism also involves FcRs (93, 101), mainly FcRIII (CD16). Cross linking of Abs that recognize an infected target cell *via* its Fab domain and the FcR on the effector cell *via* its Fc domain leads to lysis of the infected target cell subsequent to effector cell degranulation (101–105). Various immune cells such as NK cells, monocytes, macrophages, or neutrophils can induce ADCC (106). It has been suggested that ADCC participated in the 31% reduced risk of HIV infection in the RV144 trial (26). Recent studies showed that ADCC also occurred in elite controllers (107, 108). Even though a correlation between *in vitro* ADCC and protection was not demonstrated, there is evidence that ADCC might account, at least partially, for protection against SHIV/SIV challenge in the *in vivo* macaque model (109). Hence, inducing Abs with ADCC function might enhance protection and should be considered as a goal in future vaccine approaches.

## Antibody-Dependent Cell-Mediated Virus Inhibition Activity

Antibody-dependent cell-mediated virus inhibition (ADCVI) results from an interaction between an infected target cell and an effector cell expressing one or several FcγRs *via* an HIV-specific Ab. ADCVI encompasses multiple effector functions related to lytic (e.g., ADCC) and non-cytolytic (e.g., production of β-chemokines) mechanisms leading to a decreased HIV-1 infection and replication (31, 93, 95, 110).

## Role of IgA-Mediated Inhibition

In patients infected with HIV-1, a specific IgA response develops in parallel to the IgG response. Noteworthy, anti-gp41 (but not anti-gp120) IgA Abs were frequently elicited in both plasma and mucosal fluids within the first weeks after transmission. However, shortly after induction, these initial mucosal anti-gp41 Env IgA Abs rapidly declined (111). Later on, during the chronic phase, virus-specific IgA are low in both mucosa and systemic compartments (112). Interestingly, HIV-specific IgA are detected in the genital tract or the seminal fluid in seronegative (in the absence of serological HIV-specific IgG) partners of HIV-positive subjects “highly exposed persistently seronegative” (HEPS) (113–115). Mazzoli et al. first showed that IgA was detected in urine and vaginal samples from HIV-exposed seronegative individuals in the absence of IgG detection (113). In addition, virus-specific IgAs were detected in the salivary secretions of children from seropositive mothers. The presence of IgA in seronegative subjects that are in regular contact with HIV suggests its potential role in protection.

*In vitro*, IgA recapitulates for some Abs the neutralizing activity of IgG. In the case of the epitope recognized by NAb b12, the neutralizing activity of IgA was equivalent to that of the IgG (116). Furthermore, IgA displayed an additional inhibitory function involving its Fc region distinct to that of IgG. The presence of IgA at mucosal sites may involve a local activation of different immune mechanisms, such as a secretory component of IgA-mediated protection of mucus-bound IgA *in vivo* (117, 118), the aggregation of secretory IgA (119), or mechanisms that involve ADCC effectors cells expressing FcαR (such as neutrophils). Recent results from the RV144 vaccine trial demonstrated that the levels of vaccine-induced IgA in serum were associated with a lack of protection against HIV acquisition (27) and that IgA competed with IgG for ADCC activity (120). Anti-HIV IgA therefore interferes with a protective IgG function, impeding its protective potential. On contrary, it was recently shown that the anti-HIV IgA1 isotype protected macaques better than the corresponding IgA2 or IgG Ab types (3). These findings suggest that, depending on their localization and/or structure, vaccine-induced or pre-existing IgA may have either a deleterious effect by competing with potential IgG protective Abs or a significant protective effect by limiting HIV transmission at the mucosal site (3). This dual IgA activity illustrates the complexity of Ab functions, that depend on the cellular and cytokine environment.

## Mechanisms of Inhibition of HIV Cell-to-Cell Transmission

Numerous studies suggest that direct cell-to-cell transmission occurring early at the mucosal site after sexual transmission makes a major contribution to rapid HIV-1 dissemination throughout the body. This mode of transmission has important consequences for designing treatments or vaccine strategies as inhibition of this type of HIV spread is even more complex than cell-free infection. Inhibitory activity of cell-to-cell spread may depend on donor and target cells such as APC-to-T cell or T cell-to-T cell, on viral strains, multiplicity of viral infection, etc. Consequently, results may diverge and may be controversial (121, 122).

Efficient HIV transmission occurs mainly *via* the formation of virological synapses (123–126). APC-to-T cell and T cell-to-T cell transfer experiments have been used to analyze the inhibitory activity of specific anti-HIV Abs in HIV transmission. By dissecting the early steps of HIV-1 spread from DCs to autologous primary CD4 T lymphocytes, it was shown that NAbs were able to efficiently inhibit HIV-1 transmission to CD4 T lymphocytes (56). Similar inhibitory activities by Abs have also been observed by others (127–131), suggesting that HIV-1 transfer from DCs to T lymphocytes can be affected by Ab inhibition. Furthermore, Fc-mediated inhibitory activity of Abs on the infection of APCs may decrease HIV-1 transmission to surrounding T cells. This is particularly relevant for DCs and macrophages as these cells highly express FcRs (56) [reviewed in Ref. (122)]. Abs can bind FcRs and therefore inhibit HIV-1 transmission *via* FcR-mediated inhibitory activity. Some NNIAbs such as 246-D have been found to reduce significantly the percentage of infected DCs *in vitro* (56). For these Abs, a strong association was found between FcγR-specific binding capacity, inhibition of HIV-1 replication, and DC maturation. This indicates that the binding of these Abs to DCs induces the maturation of these cells, resulting in lower levels of R5 virus replication (56). However, other authors observed a drastic decrease of Ab inhibitory activity in HIV transfer conditions (51, 54, 132–134). Of note, these later studies mainly involved cell lines in *in vitro* transfer protocols and the characteristics of the cell-to-cell contact appeared to be determinant for HIV inhibition (54). Close interactions between donor/target cells and immunological synapse formation differ according to the type of cells. DC/lymphocyte crosstalk involves ICAM-1 and LFA-1 adhesion molecules and stabilize interactions (135–139) that are absent with TZM-bl cell lines (140, 141). As a result, the strength of the established synapse will influence the efficiency of HIV spread, and the subsequently inhibitory potential of Abs (54, 56). APC/lymphocyte crosstalk can also modulate the immune response (126, 139, 142–146). Close contact between cells in particular tissues need therefore to be taken into consideration when analyzing HIV transfer. However, very little is currently known about the efficacy of HIV spread and the potency of HIV-specific Abs in different tissue environments.

In addition, during chronic infection, HIV replication propagates in lymphoid organs containing numerous CD4^+^ T lymphocytes (147, 148) and cell-to-cell transmission between T cells is likely to be the most common mode of HIV-1 spread (124, 146, 149–152). HIV inhibitory activity of Abs on T cell-to-T cell transmission has been extensively studied (51, 53, 54, 133, 153–158), and variable inhibitory activities have been recorded depending on the type of T cells and virus used. These discrepancies emphasize the necessity to further investigate functional Ab activities in the context of HIV spread in tissues and lymphoid organs.

## Enhancing Antibodies

Enhancing Abs were first described as complement-mediated enhancement by Robinson et al. (159, 160). Such Abs, unlike neutralizing or inhibitory Abs, facilitate infection by HIV *in vitro* by increasing HIV titers (e.g., an increase in the number of infected cells) or by augmenting the production of infectious virus particles. Ab-dependent enhancement of HIV-1 binding and infection of certain cell types has been demonstrated in different *in vitro* protocols (160–163). However, the mechanism leading to this increase has not been clearly identified although it has been proposed that Fc–Fcγ receptor interactions or conformational changes in Env or complement receptors may play a role (164–166). The HIV-1/IgG complex is able to bind to FcRs and it could therefore be transcytosed by APCs (167). It has also been proposed that virus coated with Abs and taken up *via* the FcR on DCs may lead to enhancement by FcR-mediated transcytosis of the virus–IgG complex (163). Recently, it was shown that the binding of HIV-1-specific Abs to neonatal FcR expressed on epithelial cells could enhance transcytosis of HIV-1 at low pH (168). Since neonatal FcR was detected in areas of the genital tract that are potentially exposed to HIV-1 during sexual intercourse, this new model of Ab-dependent enhancement points to an additional mechanism by which sexual transmission of HIV-1 may be facilitated (168). However, no facilitating role of Abs has yet been demonstrated *in vivo* for HIV infection following vaccination or HIV disease progression (169). There was no evidence of an increased HIV infection among vaccine recipients in the VaxGen and RV144 phase III vaccine trials (26, 170). It should be noted that a recent study showed a correlation between the presence of a particular allele of FcγR and an increased risk of infection in a sub-group of volunteers with low risk practices (171). These results suggest a possible deleterious effect of specific HIV Abs in a subpopulation of patients with a particular FcγR genotype. Therefore, supplemental studies need to be conducted in future prophylactic vaccine trials in order to circumvent any possible deleterious enhancing effects, since vaccination obviously should avoid the induction of such Abs.

## Mechanisms of Inhibition of HIV Spread *in vivo* by Antibodies

The protective role of HIV-specific Abs has been extensively studied in various experimental models of infection, including NHP models (17, 19, 31, 172–178), and humanized mice models (14–16). The potential role of FcγR-mediated innate and adaptive immune functions in addition to neutralization has been repeatedly demonstrated in HIV protection (32, 95, 101, 110, 174, 179). Neutralizing monoclonal IgG1 b12, devoid of Fc–FcγR functions has decreased protective potential following vaginal challenge in NHPs (110). NNIAbs were able to reduce the viral load in challenged macaques without conferring complete protection (31, 32). These observations clearly indicate that, in addition to neutralization, FcγRs are important for achieving protection *in vivo*. Such effective protection observed *in vivo* suggests that HIV-specific Abs inhibit infection by cell-free virus and cell-to-cell transmission (Figure 1), both mechanisms contributing to HIV-1 replication and dissemination in the body. Interestingly, the Ab threshold necessary for sterilizing protection decreased in the animal model with decreased virus challenge (174). This further suggests that Abs may display increased potential during sexual transmission in the mucosal environment in the presence of low virus input. In this case, the balance in favor of HIV protection may be more easily achieved by vaccination, as suggested by the partial protection in the RV144 trial observed in a low risk population.

## Novel Unraveled Mechanism of Antibody Inhibition

Recently, another Ab inhibitory activity was reported that provides protection inside cells by triggering an intracellular immune response in addition to extracellular activities (180). This activity was named ADIN for antibody-dependent intracellular neutralization (181). Working with a non-enveloped virus-like adenovirus as model, it was shown that Abs that bind virus before infection were carried into the cell while attached to the virus particle. Upon escape from the endosomal compartment, these Abs remain bound to the virus allowing it to be detected by the cell. Ab-coated virions are detected by a cytosolic intracellular Ab receptor called TRIM21, which binds to IgG with a higher affinity than any other Ab receptor so far described in humans (180, 182, 183). In addition to its Ab-binding domain, TRIM21 possesses a RING domain with E3 ubiquitin ligase activity. Using this ubiquitination activity, TRIM21 flags the virion for destruction by a mechanism involving proteasomal degradation. This process is very rapid and leads to removal of the virus before transcription and translation of the viral genome, in effect clearing the cell of infection (181). Moreover, it has been shown recently that the TRIM21-mediated ability of antisera to block replication was a consistent feature of the humoral immune response in immunized mice. In the presence of immune sera and upon infection, TRIM21 also activates a proinflammatory response, resulting in secretion of tumor necrosis factor alpha (TNF-α) and interleukin-6 (IL-6) (184). These results demonstrate that TRIM21 provides a potent block to the spreading of infection and induces an antiviral state (184). However, such Ab inhibitory activity may not be relevant to HIV since HIV is an enveloped virus that is uncoated following its entry in host cells. However, if Abs against the core proteins are endocytosed during infection, they may impair later intracellular HIV replication steps. Such an intracellular mechanism may explain some unexpected association between high anti-p24 Ab concentration and decreased viral load (185, 186).

Even more intriguing, broader Ab activities have recently been proposed. The group of Nancy Haigwoog showed an increase of the specific B-cell response, following the passive transfer HIV Abs in a NHP model (187). Using the FrCas mouse retroviral model, Michaud et al. observed a protection linked to the induction of long-term B and T response, due to passive transfer of NAbs (188). This mechanism of stimulation of the adaptive response following Abs transfer was also observed following NAb therapy in infected macaques (17). Such prolonged protection by induction of adaptive immune response by Abs was already described in cancer field (189). These studies attribute an “immunogenic” role to the Abs in that they would be able to induce primary and memory responses more efficiently than free viral particles or infected cells. In this way, Abs could participate in the implementation of an adaptive response, paving the way to new fields of applications.

## Promise of HIV Antibodies in AIDS Vaccines

Currently, one of the innovating vaccination strategies would consist in developing a mucosal vaccine as an effective means of prevention against HIV sexual transmission (72). The newly identified potent bNAbs that suppress active infections and clear infected cells in humanized mice and macaques suggest that these bNAbs would effectively protect from infection (20). However, in the development of a vaccine against HIV, the possibility of inducing such NAbs have been compromised when it was discovered that they possess unusual characteristics (heavy long chain HCDR3, significant numbers of somatic mutations) that require a long maturation and makes them difficult to induce. The maturation of progenitor B-cells is unlikely to be reproduced by a short stimulation with a single immunogen (8). Vaccination strategies based on a succession of immunogens that would be able to mimic, step by step, the process of maturation, and activation of B-cell clones are currently being tested. However, this approach of vaccination has never been implemented previously and in view of the complex mechanisms that are involved, it is unclear whether it will be successful.

In view of the major, constraints linked to the *in vivo* induction of NAbs, vaccine approaches involving the optimization of inhibitory Abs, induction of additional immune mechanisms, are currently being examined. These are similar to approaches followed in cancer research, and attempt to modify the Fc region of Abs in order to increase their inhibitory activity. Moldt et al. generated by mutagenesis and by modifying the glycosylation of the Fc region, a panel of mutants of the NAb b12, which retained the neutralizing activity of the Fab region but had different affinity the FcγRs (190, 191). These Fc modifications increased the affinity of Abs for FcγRs as well as the associated *in vivo* inhibitory functions (phagocytosis, ADCC, etc.). However, no improvement of protection was observed in experimentally challenged macaques.

By inducing inhibitory Abs directly at the sites of infection (anal mucosa, genital tracts, etc.), it might be possible to limit viral replication earlier and in many target cells. New immunogens are currently being formulated in order to redirect the humoral response to mucosal sites (192). A phase 1 clinical trial has recently started (European collaborative project EuroNeut41) in order to test this new concept (193). However, these protocols are based on information gathered from the mouse model and local mucosal immune activation could not be reproduced in humans. Unfortunately, our current knowledge on how we could bring the immune response to converge toward mucosa is extremely limited and the mechanisms of action of anti-HIV Abs at mucosal sites are also poorly understood.

In addition to their central role in vaccination, Abs are also being investigated as possible therapeutic agents. Recent studies demonstrate that combinations of cocktails of two or more monoclonal Abs significantly reduced viremia in chronically infected macaques, suggesting that such therapies might be effective in humans (17, 19, 32). By combining diverse Abs properties to potentiate the protective effects of anti-HIV-specific Ab-based strategies, it might be possible to enhance what was achieved with antiviral compounds by inducing complementary inhibitory potentials gathered by Abs inhibitory functions. A combination of conventional multi-hits antiretroviral therapy with NAbs therapy might be successful and could generate revolutionary drug combinations that may lead to an HIV cure.

## Summary and Conclusion

The last decade has witnessed enormous advances in our knowledge of HIV vaccine designs and trials. Although a large number of broadly and potent NAbs have been recently discovered (Figure 2), inducing such bNAbs by vaccination is likely to be very difficult (5, 7, 10). Data from *in vivo* studies and recent findings following clinical assays have demonstrated the importance of Fc-mediated Ab-dependant mechanisms in achieving protection against HIV. Therefore, new vaccination strategies including the induction of such type of activities, in addition to NAbs, should be developed. As HIV transmission at mucosal sites involves specific HIV targets, vaccination should induce an immune response that protects all the different potential mucosal target cells (i.e., using Abs that display different inhibitory activities). Moreover, vaccination should induce Abs and B-cell responses directly at mucosal level in order to rapidly interfere with the early events of HIV infection. Almost nothing is known about the local immune induction at mucosal sites, known to be involved in induction of tolerance. Strategies to develop local immune responses should therefore be encouraged as well as specific adjuvants and immunogens active at the mucosal site leading to a strong and long-lasting response. Furthermore, the protective role of HIV-specific Abs against cell-to-cell transmission should be evaluated by analyzing the transfer of transmitted/founder HIV. It is hoped that improved understanding of HIV transmission *via* cell-free or cell-associated models and of different functionalities of HIV-specific Abs may lead to a new generation of immunogens and immunotherapeutics for the development of protective and safe vaccine approaches.

## Conflict of Interest Statement

The authors declare that the research was conducted in the absence of any commercial or financial relationships that could be construed as a potential conflict of interest.
